# Women as Doctors

**Published:** 1873-04

**Authors:** 


*Women as Doctors.*—It is less than twenty-five years since the first medical college for women began its work. Now there are several of these institutions in Europe and in this country, while the doors of many of the older colleges are open to women. There are scores of women engaged in medical studies now where they were counted by units a dozen years ago. Three hundred women have offered themselves at one college in Russia, and at Zurich sixty-three students are now engaged. The lady students there constitute one-fourth of all the matriculates. All restrictions on the admission of ladies to the lectures and laboratory practice of the Pharmaceutical Society of London have been removed, and in this country women freely attend the lectures and instructions of some of the colleges. Women physicians are members of County and State Medical Societies, and so have a representation in the American Medical Association. One phase of this medical education of women is that a call is made for female missionaries who are doctors. Three of these have already gone among the Zenanas in India, and another is soon to go to China.

